# Optimizing soil quality and rhizosphere ecology to enhance *Annona squamosa* yield and quality through water-fertilizer-mulching synergy

**DOI:** 10.1371/journal.pone.0338781

**Published:** 2025-12-12

**Authors:** Weihua Wang, Yafang Liu, Jianqi Li, Ting Bai, Weiyueheng Chen, Liyan Dong, Yibin Lu

**Affiliations:** 1 Faculty of Modern Agricultural Engineering, Kunming University of Science and Technology, Kunming, China; 2 Yuanjiang County Agricultural Machinery Management Service Center, Yuanjiang, China; 3 Faculty of Science, Kunming University of Science and Technology, Kunming, China; 4 Panzhihua Agricultural and Forestry Science Research Institute, Panzhihua, China; Dev Bhoomi Uttarakhand University, INDIA

## Abstract

To address the decline in *Annona squamosa* yield and quality in Yunnan Province resulting from inadequate irrigation and excessive fertilization, this study was focused on the interaction mechanism between rhizosphere micro ecology and fruit production of *Annona squamosa*, aiming to explore schemes to improve soil health and realize sustainable management of fruit trees. Based on field experiments conducted in 2022−2023, a three-factor, three-level orthogonal experimental design was developed (Irrigation: W1/W2/W3 for field water holding capacity is 55/75/85%; fertilization: F1/F2/F3 is 1666/2083/2500 kg·ha^-1^; mulching: A1/A2/A3 is no mulching/grass/straw mulching). The results indicated that irrigation and fertilization methods combined with mulching significantly influenced soil nutrient levels, and W2F3A1 treatment showed higher nutrient content at multiple growth stages. The activity of soil urease, phosphatase and catalase in soil reached its highest level under the W2F2A3 method. Analysis of microbial communities revealed that different treatments significantly affected the population size and diversity, with the highest microbial abundance observed under the W3F3A2 treatment. In terms of fruit quality, W2F2A3 treatment significantly increased the weight and yield of single fruit, and also stood out for soluble solids, soluble sugars, vitamin C content, and sugar-to-acid ratio. A close correlation was observed among soil nutrients, enzyme function and microbial community structure, on the one hand, and indicators of the quality of *Annona squamosa* fruit, on the other. Additionally a water-fertilizer-mulching evaluation algorithm based on PCA-GRA confirmed that W2F2A3 was the optimal solution. This study revealed the mechanism by which water, fertilizer, and mulching in orchards synergistically drive the regulation and optimization of rhizosphere micro ecology, clarified the key pathways through which straw mulching promotes nutrient cycling by regulating enzyme activity and microbial functions, and proposed the W2F2A3 optimization scheme, which provided a practical model for ecological restoration and economic benefits in green fruit tree cultivation.

## 1. Introduction

*Annona squamosa* is a drought-resistant evergreen tree that thrives in tropical lowland climates. The growth of its fruit is influenced by a number of different environmental factors [1]. Yunnan Province is located in a low-latitude inland region and has a subtropical plateau monsoon climate. Its mild temperatures throughout the year, abundant rainfall, and long period of sunshine provide favorable conditions for the growth of *Annona squamosa* [2]. As a tropical cash crop, the growth of *Annona squamosa* is highly dependent on the soil environment around its roots.The red soils of Yunnan’s hilly regions exhibit high bulk density due to intense weathering processes [3]. Their fine-grained pore structure reduces volumetric density while impeding water and gas diffusion, resulting in chronic aeration deficiencies that inhibit nutrient availability [4]. Moreover, much of Yunnan’s cultivated land lies on slopes. Rainfall triggers surface runoff and leaching, washing away soluble nutrients and clay particles. This further degrades soil structure, leading to declining soil fertility [5]. The decline in fertility reduces soil enzyme activity, affecting nutrient cycling, and lowers cation exchange capacity, leading to severe soil acidification. In addition, the mountainous terrain is rugged, and the improper use and coverage of land exacerbate soil erosion under concentrated rainfall conditions, posing a threat to the growth of *Annona squamosa* [6]. The roots of the *Annona squamosa* tree are very sensitive to soil conditions. Insufficient irrigation and excessive fertilization can lead to soil compaction, acidification, and secondary desalinization. This subsequently reduces soil aeration and permeability, diminishes soil temperature stability, and results in insufficient effective accumulated temperature [7]. This imbalance in soil nutrients not only reduces *Annona squamosa* yield but also degrades its quality. Therefore, in light of the soil fertility degradation issues confronting Yunnan’s red soils on slopes and the *Annona squamosa* industry, exploring suitable water and fertilizer management practices alongside mulching measures and their underlying mechanisms holds significant theoretical value and practical significance for improving soil quality, optimizing the soil ecological environment, and enhancing *Annona squamosa* yield and quality.

Irrigation and fertilizer management are essential components of agricultural production, serving as key practices for sustaining crop systems. The water-fertilizer combined effect is closely related to plant water and fertilizer absorption, and directly affects plant growth [8]. Studies has shown that excessive fertilization in soils with low moisture content can alter salt accumulation in the soil, causing soil salinization and inhibiting seedling growth [9]. Prolonged high soil moisture not only inhibits the respiration of fruit tree roots and reduces their absorption capacity, but also inhibits the respiration of aerobic microorganisms, thereby reducing the rate of organic matter mineralization [10]. On the one hand, lower fertilization rates lead to soil nutrient imbalances, reducing plant growth rates and thereby affecting normal crop growth [11]. On the other hand, traditional extensive irrigation and excessive fertilization increase soil compaction, secondary salinization, and inhibition of microbial activity. Seasonal droughts and concentrated rainfall have further induced soil erosion, creating a vicious cycle of ‘soil degradation-inefficient fruit trees’ [12,13]. Rational irrigation and fertilization can regulate soil moisture conditions and nutrient absorption, promote the absorption and utilization of multiple nutrients, improve soil health to achieve a good state, and increase water and fertilizer use efficiency [14]. Mulch application affects soil conditions, which in turn has a gradual and sustained impact on crop growth [15]. It helps maintain soil moisture, inhibits erosion, and reduces the impact of pesticide and fertilizer residues by limiting their leaching into groundwater, reducing runoff into surface water, and promoting microbial degradation [16]. Organic compost increases the functional diversity of soil microorganisms in the carbon and nitrogen cycle, which improves soil quality in fruit orchards in mountainous regions [17]. Straw mulching protects the soil from sudden changes in temperature, prevents weeds from growing, and conserves soil moisture, thereby increasing crop storage components and improving crop yields [18].

Although research on soil improvement through single water and fertilizer regulation or mulching measures has made progress, such as straw mulching to buffer temperature fluctuations and enhance organic matter [19] and grass mulching to improve permeability and erosion resistance [20], most studies have focused on short-term single effects and lacked an analysis of the multi-factor coupling mechanisms of water-fertilizer-mulching, especially in terms of theoretical gaps in the ecological-production synergy optimization of orchards in red soil regions. In addition, current studies neglect the prolonged effects of mulching interventions on root enzymatic functions and the activity of functional microbial communities. There are also insufficient results from field studies on the mutual influence of water, fertilizers and heat on the metabolism and nutritional value of sugar and acid in fruit. Based on this, the present study aims to elucidate the driving pathways of water-fertilizer-mulch coupling on soil nutrient cycling, enzyme activity, and microbial communities in red soil regions. It seeks to clarify the regulatory mechanisms by which multifunctional interactions influence *Annona squamosa* yield formation and quality enhancement. Consequently, an optimized management model will be established that balances soil health with efficient fruit tree production. This endeavour aims to provide theoretical underpinnings and technical paradigms for the green transformation of distinctive forestry and horticultural industries in low-latitude plateau subtropical zones.

## 2. Materials and methods

### 2.1. Overview of the experiment

The experiment was conducted in a five-year-old *Annona squamosa* orchard in Mangfei Village, Aihua Town, Yun County, Lincang City, Yunnan Province (affiliated to Yun County Kanghui Agricultural Technology Development Co., Ltd.) from March 2022 to mid-October 2023. The area is located in the southwestern part of Yunnan Province, China (100°105′309″E, 24°406′309″N), at an altitude of 1,300 meters above sea level and has a subtropical climate. The annual number of sunshine hours is 2252.3, the mean yearly temperature stands at 21.2°C, with a frost-free duration ranging from 317 to 357 days, and average yearly precipitation measures 905.6 mm. The soil density in the experimental field is 1.35 g·cm^-3^, the organic matter content is 16.12 g·kg^-1^, the pH value is 6.97, the available phosphorus is 23.8 mg·kg^-1^, the available potassium is 405 mg·kg^-1^. The experimental orchard spans more than 82 acres. It was established in 2016. The main variety planted is *Annona squamosa*. The plant spacing is 3m × 4m with 830 plants per hectare. It runs from north to south. The orchard trees are growing vigorously, with medium tree vigor. There are no diseases or pests. The growth and management status of the orchard trees is representative of this area.

### 2.2. Experimental design

The trial took place in March 2022, utilizing healthy Annona squamosa trees with uniform growth (determined by consistent biometric indices including plant height 2.5–3.0 m, and trunk diameter 6–8 cm) in the orchard as experimental subjects. A three-factor, three-level orthogonal experimental design (3^4^) was used to arrange the field experiment. The orthogonal experimental design is shown in Table 1. Three factors were set: irrigation quota (A), fertilizer application rate (B), and mulching conditions (C). The study design three levels for each factor, each replicated thrice. Thus, 27 experimental plots were utilized. The plot spans 36m^2^ (12m × 3m), and the blocks are positioned in a distribution. The three stages of irrigation are delineated as W1 intensive deficiency in irrigation (soil moisture at 55% of field’s moisture-holding capacity), low deficit irrigation W2 (soil moisture at 75% of field’s moisture-holding capacity); and adequate irrigation W3 (soil moisture at 85% of field’s moisture-holding capacity), soil moisture levels were recorded biweekly. The irrigation’s upper threshold was established at the pre-defined three levels, with the lower boundary set by the soil moisture reading prior to each irrigation event. The formula for calculating irrigation water volume is as follows:

**Table 1:** Orthogonal test scheme.

Treatment	Factor		
	Soil moisture	Rate of fertilizer application (kg·ha^-1^)	Mulching
W1F1A1	W1(55%FC)	F1(1666)	A1(No-Mulching)
W1F2A2	W1(55%FC)	F2(2083)	A2(Grass Mulching)
W1F3A3	W1(55%FC)	F3(2500)	A3(Straw Mulching)
W2F1A2	W2(75%FC)	F1(1666)	A2(Grass Mulching)
W2F2A3	W2(75%FC)	F2(2083)	A3(Straw Mulching)
W2F3A1	W2(75%FC)	F3(2500)	A1(No-Mulching)
W3F1A3	W3(85%FC)	F1(1666)	A3(Straw Mulching)
W3F2A1	W3(85%FC)	F2(2083)	A1(No-Mulching)
W3F3A2	W3(85%FC)	F3(2500)	A2(Grass Mulching)


M=10γH(θmax−θ0) (1)

Where M represents the irrigation water volume (mm); γ denotes the density of the soil at the corresponding moisture content level, at a density of 1.3 g·cm⁻^3^; H denotes the soil moisture depth within the observed layer, taken as 0.8 m; θ_max_ denotes the maximum threshold for irrigation water (%); θ_0_ represents the initial soil moisture percentage prior to irrigation.

As shown in Table 1, the W1F1A1 treatment (55% ETc irrigation, 1666 kg·hm^-2^ fertilizer application, no mulching) set all factors at their minimum levels, serving as a control for assessing the incremental effects of irrigation, fertilizer and mulching measures.

The fertilization rates were categorized as F1 (1666 kg·ha^-1^), F2 (2083 kg·ha^-1^), and F3 (2500 kg·ha^-1^). The trial fertilizer consisted of a commonly used water-soluble formulation intended for use on fruit trees (15 N – 15 (P_2_O_5_)-15 (K_2_O), Xuelv Feng, produced by Saigute Biotechnology Co., Ltd.) Three mulching treatments are A1 (unmulched), A2 (utilizing grass), and A3 (utilizing straw). In this experiment, white clover was used as a cover crop to suppress weeds through dense growth. No manual weeding was performed throughout the experiment, and the cover crop’s competitive effect maintained a weed-free state in the experimental area. The seeds we planted were pure white clover, with a generous spread of 50 kg·hm^-2^, and straw mulching utilized entire harvested corn stalks. Uniformly applied across the plot surface with a consistent 15 cm thickness along the rows.

**Fig 1:**
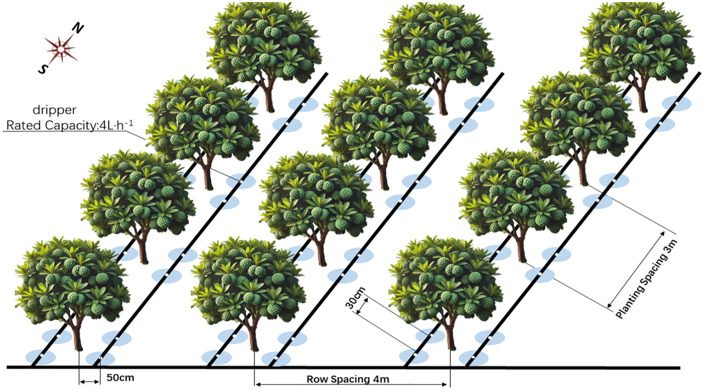
The layout of the plot and the drip tape for *Annona squamosa.*

The vegetative development of *Annona squamosa* occurred through four distinct stages: the initial phase of bloom bud development spans from March 15th to May 5th, followed by the blooming and fruiting period from May 6th to June 15th. The third stage, where the fruits swell, runs from June 16th to August 15th. Finally, the fruits reach full maturity between August 16th and October 5th. Parallel irrigation strips were installed alongside each row of planting. The irrigation collar was set 50 cm away from the tree’s base, utilizing a 4 L·h^-1^ flow rate through a dripper spaced 30 cm apart, and four drippers per tree facilitated combined water and fertilizer supply through integrated irrigation (Fig 1). There are 5 *Annona squamosa* trees in each plot. Three of the trees with the best growth performance were selected for sampling. All agricultural management practices in each plot were identical, apart from the soil treatments.

### 2.3. Soil and *Annona squamosa* yield and quality analysis

#### 2.3.1. Sampling of soil around *Annona Squamosa* roots.

Soil samples were collected on 25 March, 20 May, 27 July and 26 September 2022, and on 29 March, 8 June, 4 August and 29 September 2023. Three well-developed *Annona squamosa* trees were selected within each plot. Soil samples were collected using a soil auger at a depth of 25 cm from the trunk, randomly selecting four positions at 10 cm intervals. Surface debris was removed, with a total sampling depth of 40 cm. After removing debris, store the sampled soil in 10 cm sections. On the sampling day, sieved a portion of the fresh soil samples and store them in a 4°C refrigerator for microbial plate testing. Another portion was preserved with dry ice and transported to the laboratory for analysis. The remaining soil samples were sieved after natural air-drying for future use.

#### 2.3.2. Measurement of soil nutrients.

Soil nitrate nitrogen (NO₃ ⁻ -N) content is determined using ultraviolet spectrophotometry at a wavelength of 210 nm on KCl leachate with a soil-to-water ratio of 5:1 [21]; soil available phosphorus (AP) content is measured using a spectrophotometer to obtain the absorbance value, with the available phosphorus content calculated according to the standard curve [22]; soil available potassium (AK) content was determined by leaching with 1 mol·L ⁻ ¹ NH₄OAc, with the leachate analyzed using flame photometry [22].

#### 2.3.3. Measurement of soil enzyme activity and microorganisms.

Soil urease activity is determined using the sodium phenolate-sodium hypochlorite colorimetric method, with absorbance measured at 578 nm using a UV spectrophotometer. The activity is expressed as the mass (mg) of NH₃-N released from 1 g of soil after 24 hours [22]; Soil phosphatase activity is measured using the sodium phosphate-sodium benzoate colorimetric method. Absorbance is determined at 660 nm using a UV spectrophotometer, expressed as the amount of phenol hydrolyzed from 1 g of air-dried soil over 24 hours (mg) [22]; soil catalase levels were assessed via potassium permanganate titration, and the volume (ml) of 0.1 N potassium permanganate consumed by the soil after shaking for 20 minutes was reported as the result [23].

Illumina MISEQ sequencing technology was used to characterize microbial assemblage within the *Annona squamosa* rhizosphere soil, and the specific method is described [24]. Weigh 0.5g of homogenized soil sample and extract total genomic DNA using the E.Z.N.A.® Soil DNA Kit (Omega Bio-Tek, USA). Following extraction, DNA concentration and purity were assessed using a NanoDrop™ 2000 UV-Vis Spectrophotometer (Thermo Fisher Scientific Inc., Waltham, MA, USA), whilst DNA integrity was verified via 1% agarose gel electrophoresis. For 16S rDNA sequencing, forward and reverse primers 341F (5’-CCTAYGGGRBGCASCAG-3’) and 806R (5’-GGACTACNNGGGTATCTAAT-3’) were employed for PCR amplification. The 25 μL reaction mixture comprised 12.5 μL 2 × Taq Plus Master Mix, 1 μL forward and reverse primers (10μmol/L), 2 μL template DNA, and 8.5 μL sterile water. The reaction underwent initial denaturation at 95°C for 5 minutes, followed by 30 cycles of 95°C for 30 seconds, 55°C for 30 seconds, and 72°C for 45 seconds, concluding with a final extension at 72°C for 10 minutes. Sequencing data were analyzed from soil samples, and 44418 bacterial OTUs were obtained according to OTU clustering, and then diversity analysis was performed on each group to compare species diversity within each microbial community.

#### 2.3.4. Measurement of yield quality.

Once the fruit has ripened, selected three *Annona squamosa* trees from each treatment group and harvest three *Annona squamosa* fruits from each tree. Fruit weight was measured by weighing technique. In the ripe season of *Annona squamosa*, assessed the fruit yield from chosen *Annona squamosa* trees per treatment. Then, compute the yield per hectare using the actual measurements taken. After harvesting, fruits were kept at room temperature to soften before measuring its quality indicators. Conventional techniques were employed to assess titratable acidity and soluble solids [25]; the anthrone colorimetric method was used to measure soluble sugar levels [26]; vitamin C levels were ascertained through spectrophotometric analysis [27]; the sugar-to-acid ratio is the ratio of soluble sugar to organic acid.

### 2.4. PCA-GRA algorithm

#### 2.4.1. Principal Component Analysis (PCA).

Twelve relevant traits of soil nitrate, available phosphorus, available potassium, urease, phosphatase, catalase, *Annona squamosa* single fruit weight, yield, soluble solids, soluble sugar, sugar-acid ratio and vitamin C content were analyzed [28].

Standardize the data:


$$Z_{ij}=\frac{X_{ij}-\mu_{j}}{\sigma_{j}}$$ (2)

Where $\mu_{j}$ denotes the average of column j, while $\sigma_{j}$ signifies the standard deviation for column j.

Calculate the covariance matrix:


$$C=\frac{n}{n-1}Z^{T}Z$$ (3)

Perform feature decomposition on C:


$$C=VAV^{T}$$ (4)

A denotes the diagonal matrix of eigenvalues, with V holding the corresponding eigenvectors.

Select the main factors and determine their respective contribution percentages:


$$D_{k}=\frac{\lambda_{k}}{\sum_{i=1}^{p}\lambda_{i}}$$ (5)

Typically, the initial m principal components are chosen if their cumulative contribution exceeds 85%. Calculate principal component scores:


$$F=ZV_{m}$$ (6)

$V_{m}$ is a matrix composed of the first m eigenvectors.

Before calculating the data, it is necessary to conduct statistical testing of the test data, and select the KMO test method to test whether the data can be evaluated in relevance [29].


$$KMO=\frac{\sum_{i\neq j}r_{ij}^{\text{2}}}{\sum_{i\neq j}r_{ij}^{\text{2}}+\sum_{i\neq j}p_{ij}^{\text{2}}}$$ (7)

Where $p{\mathrm{\backslash nolimits}}_{ij}^{2}$ is the square of the correlation coefficients of each variable, and $r{\mathrm{\backslash nolimits}}_{ij}^{2}$ is the square of the partial correlation coefficient. The KMO value is close to 1, which means that the data is very suitable for factor analysis. On the contrary, if the partial correlation coefficient is large, the KMO value will become smaller, indicating that the data is not suitable for factor analysis.

#### 2.4.2. Grey Relational Analysis (GRA).

The gray system-based GRA takes into account the 9 treatments in the experiment. The optimal treatment scheme for comprehensive *Annona squamosa* is calculated according to the following steps [30].

The steps of the GRA algorithm are as follows:

Provide reference sequence $x_{0}$: the ideal optimal scheme.

Comparison Sequence $x_{i}$: Data Arrangement of Each Protocols to be Evaluated

Data Standardization Processing:


$$x_{i}^{\prime }(k)=\frac{x_{i}(k)-\min x_{i}(k)}{\max x_{i}(k)-\min x_{i}(k)}$$ (8)

Determine the correlation coefficient to indicate the extent of association between the comparative sequence and the benchmark sequence at each interval:


$$\xi_{i}(k)=\frac{\underset{i}{\min}\underset{k}{\min}\left|{x^{\prime }}_{0}(k)-{x^{\prime }}_{i}(k)\right|+\rho \underset{i}{\max}\underset{k}{\max}\left|{x^{\prime }}_{0}(k)-{x^{\prime }}_{i}(k)\right|}{\left|{x^{\prime }}_{0}(k)-{x^{\prime }}_{i}(k)\right|+\rho \underset{i}{\max}\underset{k}{\max}\left|{x^{\prime }}_{0}(k)-{x^{\prime }}_{i}(k)\right|}$$ (9)

ρ is the resolution ratio, typically set at 0.5.

Calculate the correlation coefficient:


$$r_{i}=\frac{\text{1}}{n}\sum_{k=1}^{n}\xi_{i}(k)$$ (10)

Based on the above algorithm, the optimal evaluation scheme can be obtained by correlation ranking analysis.

#### 2.4.3. Water and fertilizer coverage evaluation algorithm based on PCA-GRA.

PCA clustered the twelve variables into distinct groups based on their loadings on PC1, PC2, and PC3. The analyzed variables, logically grouped as soil nutrients (nitrate nitrogen, available phosphorus, available potassium), soil enzymes (urease, phosphatase, catalase), and fruit traits (yield, single fruit weight, soluble solids, soluble sugar, sugar-acid ratio, vitamin C), showed clear groupings [31]:


$$W{\mathrm{\backslash nolimits}}_{k}=\frac{PC{\mathrm{\backslash nolimits}}_{k}}{\sum {\mathrm{\backslash nolimits}}_{k-1}^{m}PC{\mathrm{\backslash nolimits}}_{k}}$$ (11)

where k = 1, 2, m (m is the number of principal components retained, satisfying the cumulative contribution rate > 85%), which is the percentage of variance explained for each principal component, and $\sum {\mathrm{\backslash nolimits}}_{k-1}^{m}W{\mathrm{\backslash nolimits}}_{k}=1$.


$$W{\mathrm{\backslash nolimits}}_{ij}=\frac{W{\mathrm{\backslash nolimits}}_{k}}{n{\mathrm{\backslash nolimits}}_{k}}$$ (12)

where $W{\mathrm{\backslash nolimits}}_{ij}$ is the weighted weight of the ith treatment jth variable, $W{\mathrm{\backslash nolimits}}_{k}$ is the weight of the kth principal component, and $n{\mathrm{\backslash nolimits}}_{k}$ is the number of variables contained in the kth principal component.

Based on the above calculated PCA weight and GRA correlation degree, the PCA – GRA weighted correlation degree is obtained as follows:


$$r{\mathrm{\backslash nolimits}}_{i}=\sum {\mathrm{\backslash nolimits}}_{j=1}^{n}W{\mathrm{\backslash nolimits}}_{ij}\times \xi {\mathrm{\backslash nolimits}}_{ij}$$ (13)

Where $r{\mathrm{\backslash nolimits}}_{i}$ is the PCA-GRA weighted correlation degree, and then the weighted correlation degree is converted into a comprehensive score.


$$S{\mathrm{\backslash nolimits}}_{i}=\frac{r{\mathrm{\backslash nolimits}}_{i}}{\max(r{\mathrm{\backslash nolimits}}_{i})}$$ (14)

Where $S{\mathrm{\backslash nolimits}}_{i}$ is the PCA-GRA composite score of the ith treatment, and $\max(r{\mathrm{\backslash nolimits}}_{i})$ is the largest weighted correlation among all treatments.

### 2.5. Data processing and analysis

Data were analyzed using Excel, SPSS 23.0, and MATLAB 2020b; Origin 2021 was used for graphing and correlation analysis.

## 3. Results

### 3.1. Effects of different water-fertilizer-mulching treatments on nutrient content in the rhizosphere soil of *Annona squamosa*

**Fig 2:**
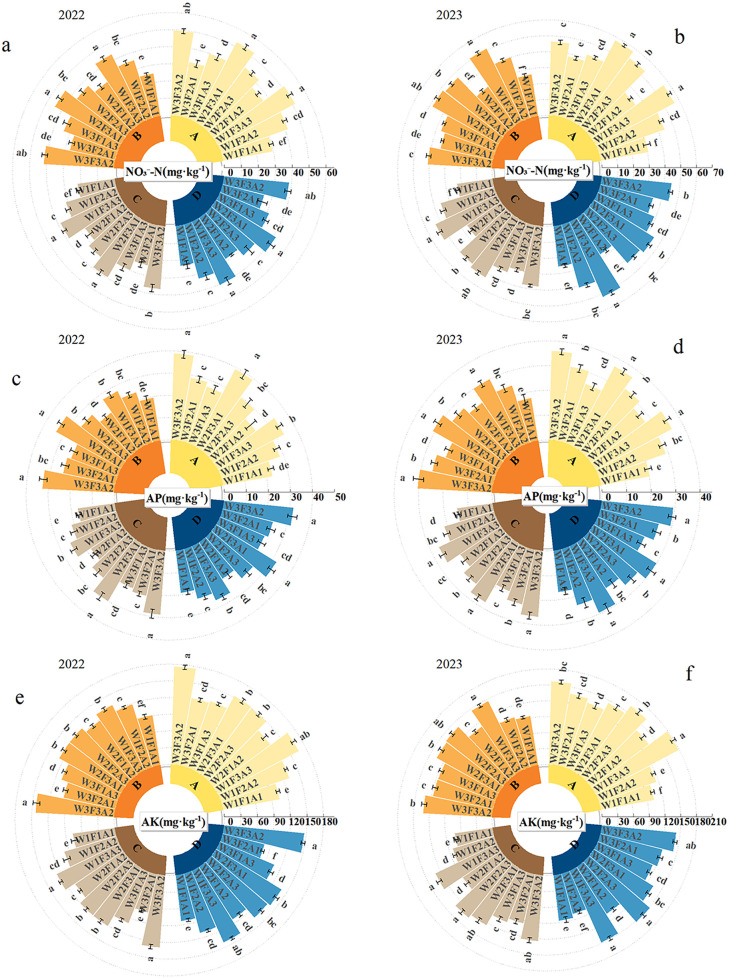
Changes in soil nutrient content during the growth period of *Annona squamosa* under different water-fertilizer-mulching treatments (2022-2023).

The contents of NO₃ ⁻ -N, AK, and AP during the four growth stages of *Annona squamosa* in 2022 and 2023 are shown in Fig 2. Under different treatments, the content of NO₃ ⁻ -N, AK, and AP in the soil initially showed a declining trend in both years, but then increased with the delayed growth of *Annona squamosa*, and the amount was higher during the differentiation phase of *Annona squamosa* flower buds. Different treatments had significant effects on nutrient content in rhizosphere soil of *Annona squamosa* (P < 0.050). The data in Fig 2a and Fig 2b show that the NO₃ ⁻ -N content decreased the most during flowering and fruit setting, with 14%−17% in 2022 and 10%−17% in 2023. At the flower bud differentiation stage, the NO₃ ⁻ -N content in the W2F3A1 treatment was the highest, with increases of 78% and 89% in 2022 and 2023, respectively, compared with the W1F1A1 treatment. In 2022, the highest NO₃ ⁻ -N content was observed during flowering and fruit formation and fruit growth and ripening in the case of W2F3A1 treatment, which represents an increase of 77.6%, 77.7%, and 77.4% compared to treatment with W1F1A1; In 2023, treatment with W1F3A3 recorded the highest content, with an increase of 93%, 90%, and 103%. The contents of AP during the bud differentiation period, flowering and fruit formation period, fruit filling period and maturity period in 2022 and 2023 were higher in the case of the W2F3A1 treatment, with an increase of 73% to 89% and 73% to 88%, in comparison to the W1F1A1 treatment (Fig 2c, 2d). The content of AK during the bud development phase, flowering and fruit formation stage, fruit filling period, and maturity stage in 2022 was the highest under the W3F3A2 treatment, with an increase of 69% to 87% compared to treatment with W1F1A1; in 2023, the W1F3A3 treatment showed the highest increase, ranging from 74% to 92%. The higher decrease in AK was observed during the fruit filling period, with a 15% decrease in 2023 compared to the W1F1A1 treatment. The most significant (p < 0.050) reduction in alternate treatments was detected during the bloom and fruiting initiation phase, with a decrease of 14% to 19% in 2022 and 14% to 17% in 2023 (Fig 2e and 2f).

### 3.2. Soil enzyme activity in the rhizosphere of *Annona squamosa*

**Fig 3:**
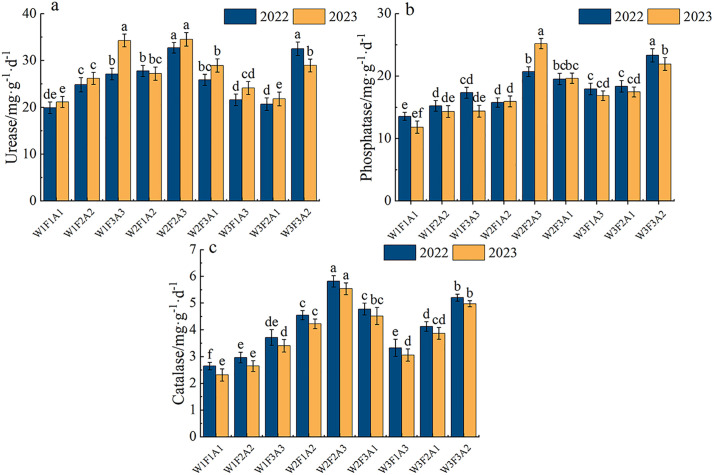
Enzyme activities under different water-fertilizer-mulching treatments in 2022 and 2023.

Fig 3 shows that water and fertilizer management and mulching significantly (P < 0.050) affected the average soil enzyme activity throughout the entire experimental period of the *Annona squamosa*. Compared with W1F1A1 treatment, urease, phosphatase and catalytic activity increased in most cases. As shown in Fig 3a, both 2022 and 2023 exhibited high urease activity under the W2F2A3 treatment, with values of 32.72 mg·g^-1^·d^-1^ and 34.55 mg·g^-1^·d^-1^, respectively. Under the F2 fertilization level, the urease activity showed an increasing trend and then a decline, depending on the amount of irrigation water and mulching technique. The urease activity reached its peak in the W2F2A3 treatment. Among them, in 2022 and 2023, compared to the W3F2A1 treatment, W2F2A3 treatment resulted in faster growth of the urease activity, increasing by 58.1% and 58.3%, respectively. The phosphatase activity is shown in Fig 3b. In 2022, it registered the peak with the W3F3A2 treatment, at 23 mg·g^-1^·d^-1^. In 2023, it reached the maximum under the W2F2A3 treatment, at 25.22 mg·g^-1^·d^-1^. Compared with the W2F2A3 treatment in 2022, it significantly (P < 0.050) increased by 22%. Under different fertilization and mulching treatments, when subjected to the mild water deficit irrigation condition of W2, the activity of catalase was significantly increased contrasted to the adequate irrigation of W1 treatment. Catalase activity in the soil remained at the highest level in treatment W2F2A3 both in 2022 and 2023, being 6.45 mg·g^-1^·d^-1^ and 6.09 mg·g^-1^·d^-1^ respectively. Under W1F1A1 treatment, enzyme activity was at its lowest level, with values of 2.94 mg·g ⁻ ¹·d ⁻ ¹ and 2.65 mg·g ⁻ ¹·d ⁻ ¹ (Fig 3c).

### 3.3. Soil microorganisms in the rhizosphere of *Annona squamosa*

As shown in Table 2, the Chao1 index was the highest in the W1F2A2 treatment among the soil samples under different treatments. The Simpson index showed little difference among all treatments; the Shannon index was the highest in the W1F2A2 treatment; the PD_whole_tree index showed higher values for the W1F3A3 and W2F2A3 treatments; and the microbial coverage index was all greater than 0.999.

**Table 2:** The α -diversity index of soil microorganisms in the rhizosphere of *Annona squamosa* in Yun County from 2022 to 2023 under different water and fertilizer mulching conditions.

Treatment	Chao1	Simpson	Shannon	PD_whole_	Coverage
W1F1A1	2144.93 ± 1417.84ab	0.983 ± 0.023a	8.56 ± 2.00b	13.16 ± 2.25d	0.9998 ± 0.0001a
W1F2A2	2968.07 ± 309.38a	0.997 ± 0.001a	10.24 ± 0.20a	19.52 ± 1.16c	0.9995 ± 0.0001b
W1F3A3	2903.1 ± 106.73a	0.998 ± 0.000a	10.21 ± 0.11a	25.55 ± 0.47a	0.9994 ± 0.0004b
W2F1A2	2776.75 ± 196.47ab	0.998 ± 0.000a	10.19 ± 0.12a	20.12 ± 0.5bc	0.9994 ± 0.0001b
W2F2A3	2397.69 ± 241.27ab	0.998 ± 0.000a	9.92 ± 0.23a	24.94 ± 1.28a	0.9994 ± 0.0001b
W2F3A1	2857.12 ± 70.79ab	0.998 ± 0.000a	10.20 ± 0.02a	21.21 ± 1.1bc	0.9995 ± 0.0001b
W3F1A3	1755.28 ± 292.19b	0.988 ± 0.006a	8.36 ± 0.63b	21.68 ± 0.87b	0.9996 ± 0.0002ab
W3F2A1	2781.19 ± 333.92ab	0.997 ± 0.001a	9.97 ± 0.3a	21.82 ± 1.44b	0.9996 ± 0.0001ab
W3F3A2	2878.22 ± 138.81ab	0.998 ± 0.000a	10.4 ± 0.08a	19.41 ± 1.05c	0.9997 ± 0.0002ab

**Fig 4:**
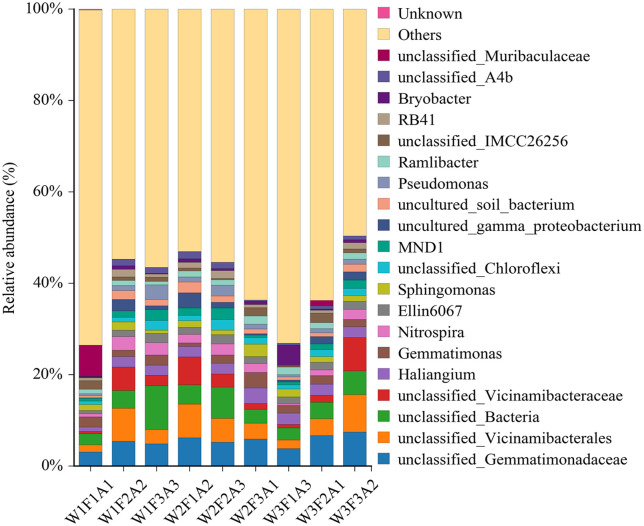
Histogram of microbial species distribution in rhizosphere soil of *Annona squamosa* under different water and fertilizer mulching treatments from 2022 to 2023.

As shown in Fig 4, the relative abundances of soil microbial species varied significantly under different treatments. Among them, the relative abundance of soil microorganisms was highest under the W3F3A2 treatment condition. The mulching treatment has a significant impact on microbial species abundance. Under A1 and A2 treatments, the cumulative relative abundance of dominant microbial species (accounting for more than 1% of the total community in each sample) is higher than that under A3 treatment. The relative species abundance of *unclassified_Gemmatimonadaceae* and *unclassified_Vicinamibacterales* was higher in all soil samples, followed by *unclassified_Bacteria*. Relative species richness of the *unclassified_Vicinamibacteraceae* in W1F2A2, W2F1A2, and W3F3A2 treatments was more obvious than that in other treatments. *Unclassified_Muribaculaceae* species richness was greater in W1F1A1, whereas *Bryobacter* richness was elevated in W3F1A3.

**Fig 5:**
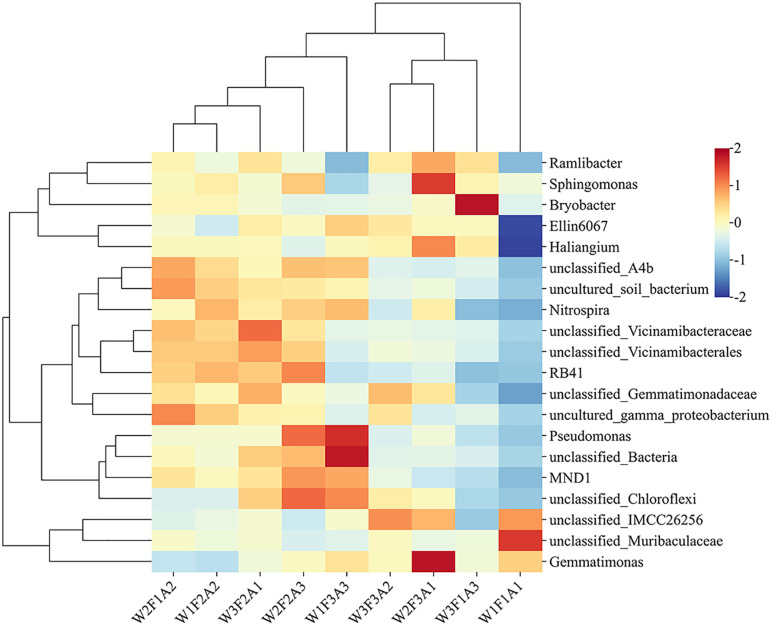
Heat map of microbial abundance in rhizosphere soil under different water and fertilizer mulching from 2022 to 2023.

The heat map of the relative abundance of different microorganisms is shown in Fig 5. The relative abundance of *unclassified_Chloroflexi*, *Pseudomonas* and *RB41* was higher under W2F2A3 treatment, while the relative abundance of *unclassified_IMCC26256*, *unclassified_Muribaculaceae* and *Haliangium* was low. In contrast, the relative abundance of *unclassified_Muribaculaceae* and *unclassified_IMCC26256* was the highest in the W1F1A1 treatment.

### 3.4. Variations in *Annona squamosa*’s quality and yield with various treatments

**Fig 6:**
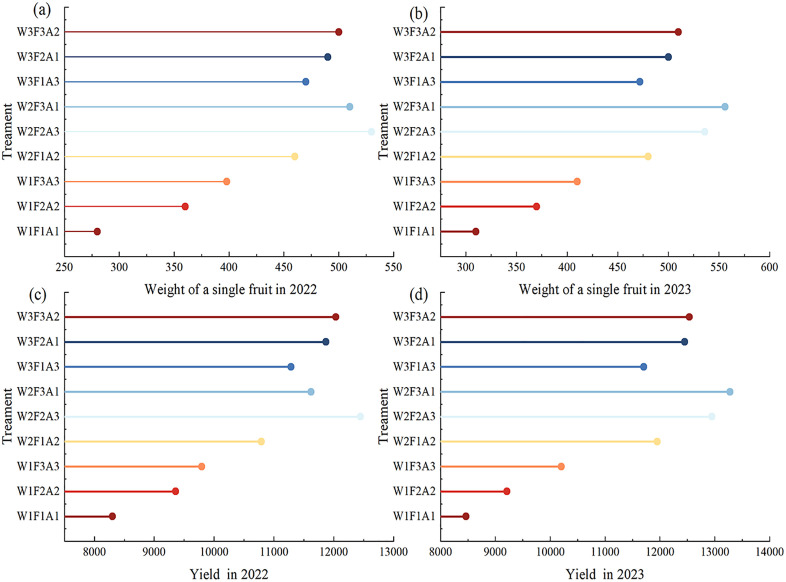
Yield and single fruit weight of *Annona squamosa* under different treatments.

Fig 6 shows the changes in the fruit quality of the *Annona squamosa* over two years. Fruit yield indicators were significantly (P < 0.050) influenced by irrigation, fertilizer, and mulching. In 2022 and 2023, the weight of single *Annona squamosa* fruit initially increased and then decreased with irrigation increased, reaching the highest values under the treatments of W2F2A3 and W2F3A1 (530 g and 556 g), respectively. Within two years, the fertilization amounts of the three gradients (1666/2083/2500 kg·ha^-1^) were significantly positively correlated with the single fruit weight. In 2022, the weight of a single fruit in the treatment F3 was 14% higher than that in F1 and 2% higher than that in F2. In 2023, it increased by 15% and 5%, respectively. The highest yield was 12450 kg·ha^-1^ under W2F2A3 treatment in 2022 and 13280 kg·ha^-1^ under W2F3A1 treatment in 2023. In 2022 and 2023, the average increase in *Annona squamosa* yield under A3 conditions exceeded that of A1 and A2 measures, with an average improvements of 6.1% and 4.8% compared with the treatment under A1 and A2 measures, respectively. Table 3 revealed the W2F2A3 treatment yielded the highest soluble solids concentration in *Annona squamosa* in both 2022 and 2023, at 28.9% and 28.8%, respectively. The soluble sugar content initially showed an increased trend, but decreases once irrigation water reaches a higher level. Both 2022 and 2023 achieved the highest value (194.69 mg g^-1^ and 195.79 mg g^-1^) under the W2F2A3 treatment. And among them, mulching treatment A3 had a significant impact on the other two mulching treatment. The W2F2A3 treatment yielded the maximum sugar-acid ratio over the two-year period, which was 10.6% and 10.9% higher than that of the W1F1A1 treatment respectively in the two years. The mulching treatment A3 reduced the acidity of *Annona squamosa* fruit while increasing their sugar content, meaning sugar-acid proportion over the subsequent two years for A3 was 2.29%–2.31% higher than that for A1 and A2. The W2F2A3 regimen yielded the highest vitamin C concentration after two years, exceeding the W1F1A1 level by 4% and 7%.

**Table 3:** Effects of different treatments on the nutrient composition of *Annona squamosa.*

Year	Treatment	the soluble solids (%)	soluble sugar (mg·g^-1^)	sugar-acid ratio	Vitamin C content (mg·100mL^-1^)
2022	W1F1A1	20.32 ± 0.05g	167.56 ± 1.03e	66.5 ± 0.05g	36.61 ± 0.02d
	W1F2A2	22.41 ± 0.05f	173.89 ± 2.04c	67.8 ± 0.04f	36.82 ± 0.06c
	W1F3A3	23.62 ± 0.06e	177.32 ± 1.24bc	69.87 ± 0.07d	37.24 ± 0.05ab
	W2F1A2	26.58 ± 0.04c	182.32 ± 1.21b	71.06 ± 0.07 cd	37.56 ± 0.05ab
	W2F2A3	28.94 ± 0.05a	194.69 ± 1.33a	73.56 ± 0.55a	37.92 ± 0.05a
	W2F3A1	27.56 ± 0.04b	183.78 ± 1.25b	72.49 ± 0.08b	37.62 ± 0.04a
	W3F1A3	23.46 ± 0.03e	174.45 ± 1.56c	70.33 ± 0.12d	37.11 ± 0.02bc
	W3F2A1	25.58 ± 0.07d	178.69 ± 1.67b	69.96 ± 0.05d	37.32 ± 0.04ab
	W3F3A2	22.94 ± 0.05f	169.69 ± 1.35d	68.77 ± 0.04e	36.95 ± 0.04c
2023	W1F1A1	21.12 ± 0.06fg	170.74 ± 1.23f	67.36 ± 0.07g	35.82 ± 0.04 cd
	W1F2A2	21.91 ± 0.07f	176.59 ± 1.14de	68.88 ± 0.06f	36.30 ± 0.06c
	W1F3A3	23.12 ± 0.07d	179.66 ± 1.09d	69.97 ± 0.08f	36.85 ± 0.05bc
	W2F1A2	25.46 ± 0.05c	187.65 ± 1.36b	72.36 ± 0.07c	37.65 ± 0.05ab
	W2F2A3	28.76 ± 0.04a	195.79 ± 1.44a	74.67 ± 1.55a	38.23 ± 0.05a
	W2F3A1	26.56 ± 0.04b	182.58 ± 1.52c	73.59 ± 0.08b	37.92 ± 0.04a
	W3F1A3	22.96 ± 0.06e	176.45 ± 0.09de	71.34 ± 0.18d	37.24 ± 0.03b
	W3F2A1	24.68 ± 0.07c	172.69 ± 0.08e	70.23 ± 0.08e	37.57 ± 0.04ab
	W3F3A2	21.57 ± 0.06f	170.69 ± 1.03f	71.11 ± 0.07d	36.75 ± 0.04bc

### 3.5. *Annona squamosa* correlation and PCA-GRA assessment

**Fig 7:**
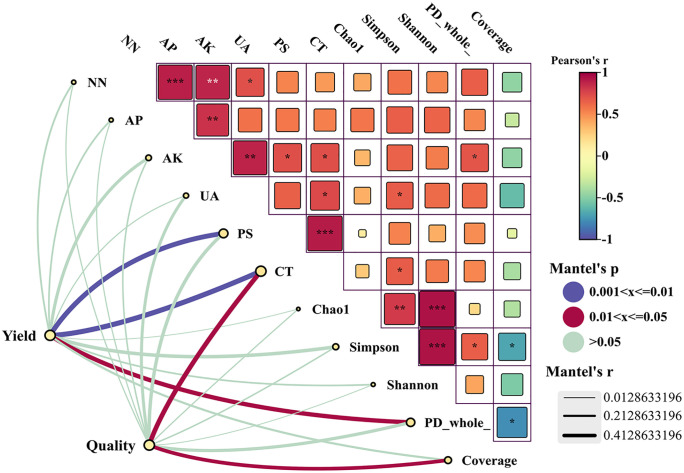
Correlation analysis between yield, quality parameters, soil environment, and growth in *Annona squamosa.* (Note: NO₃ ⁻ -N: nitrate nitrogen, AP: available phosphorus, AK: available potassium, UA: urease activity, PS: phosphatase activity, CT: catalase activity, Chao1: species richness index, Simpson: community richness index, Shannon: species evenness index, PD_whole_: community phylogenetic diversity index, Coverage: sequencing depth index. *Characterizes the significance of the correlation, * for P < 0.050, ** for P < 0.010, *** for P < 0.001, and no for P > 0.050.).

As shown in Fig 7, it is the Mantel test relationship of the yield and quality of *Annona squamosa* with various environmental indicators of the soil. The analysis revealed significant positive correlations (P < 0.001) between soil NO₃ ⁻ -N and AP contents, AP content and catalase activity, and both Chao1 and Shannon indices. In addition, the correlations demonstrated a negative correlation with the microbial coverage index, Simpson microorganism index for soil, and PD_whole_tree (P < 0.050). Soil NO₃ ⁻ -N concentrations were significantly (P < 0.050) positively correlated with urease function. Notably, elevated AK concentrations in the soil were linked to a rise in soil phosphatase enzymatic activity, catalase activity and PD_whole_tree index (P < 0.050), and soil AK levels showed a highly significant (P < 0.010) positive link to urease activity.

As shown in Table 4, the variance interpretation analysis of each principal component is set at 85%. PC1 mainly refers to the two variables of soil nutrient index and soil enzyme activity index. PC2 mainly refers to the yield and quality variable of *Annona squamosa*. PC3 is the principal component with the least contribution, which is composed of secondary environmental variables and special nutrient variables.

**Table 4:** Interpretation rate of variance of each component under principal component analysis.

Principal Component	eigenvalue	Variance Interpretation Rate (%)	Cumulative Variance Interpretation Rate (%)	Whether to retain or not
PC1	8.018	67.57	67.57	Yes
PC2	1.909	15.91	83.47	Yes
PC3	1.082	9.02	92.49	Yes
PC4	0.482	4.02	96.51	No
PC5	0.176	1.47	97.98	No
PC6	0.127	1.06	99.04	No
PC7	0.098	0.81	99.85	No
PC8	0.018	0.15	100.00	No
PC9	0.000	0.00	100.00	No
PC10	0.000	0.00	100.00	No
PC11	0.000	0.00	100.00	No
PC12	0.000	0.00	100.00	No

After calculating the test data, the KMO value is calculated to be 0.6863, and the KMO value of each variable is also calculated as shown in Table 5. According to the calculated data, it is indicated that our data to be tested is suitable for factor analysis and other discriminant analysis methods. The load matrix for each principal component is obtained as follows.

**Table 5:** Core load variables and KMO test values of PC1 and PC2 under the synergistic effect of water and fertilizer covering.

index	PC1	PC2	PC3	KMO
NO₃ ⁻ -N	0.228	−0.508	0.336	0.0776
AP	0.227	−0.470	0.371	0.0796
AK	0.290	−0.337	−0.305	0.0837
UA	0.252	−0.377	−0.637	0.0867
PS	0.304	0.089	−0.075	0.0775
CT	0.324	0.074	−0.035	0.0798
FW	0.317	0.153	−0.029	0.0880
YD	0.307	0.196	0.174	0.0891
TSS	0.293	0.258	0.072	0.0871
FSS	0.249	0.166	−0.386	0.0825
SAR	0.333	0.173	0.191	0.0848
VC	0.313	0.257	0.150	0.0836

Note: FW: single fruit weight; YD: Output; TSS: soluble solids; FSS: soluble sugar; SAR: sugar-acid ratio; VC: vitamin C

**Fig 8:**
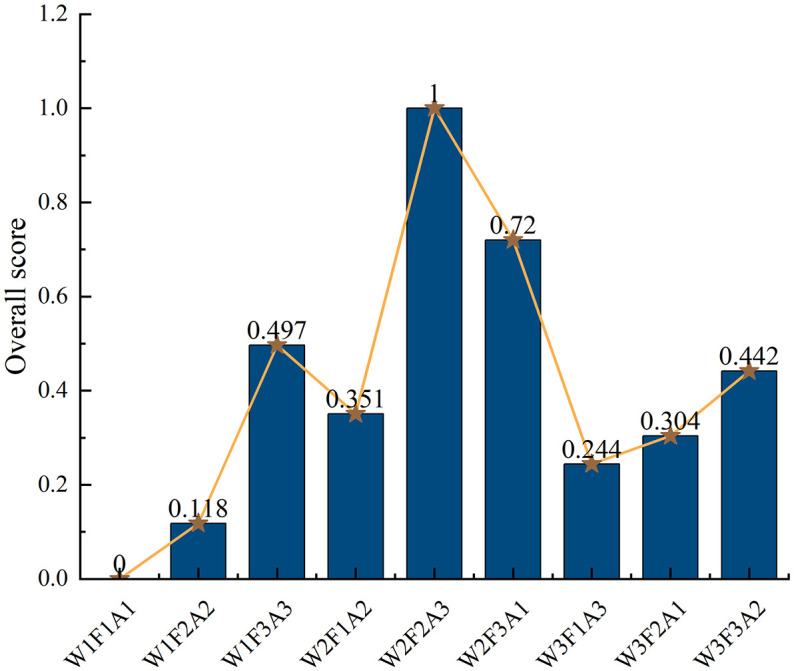
Results of PCA-GRA-based water and fertilizer mulching evaluation algorithm for each indicator.

The aggregate mean values obtained by the water and fertilizer coverage evaluation algorithm based on PCA-GRA are shown in the figure. From the data in Fig 8, comprehensive evaluation analysis shows that the average value of aggregation increased first and then decreased, and then increased and then decreased with the progress of treatment, and within this fluctuating trend. Specifically, the aggregated average of water-fertilizer-coverage evaluation under W2F2A3 reached the optimal solution (1.00), which was significantly higher than the aggregated average of other treatments. Among them, the ranking of each treatment is W2F2A3>W2F3A1 > W1F3A3 > W3F3A2 > W2F1A2 > W3F2A1 > W3F1A3 > W1F2A2 > W1F1A1.

## 4. Discussion

### 4.1. The impact of water and fertilizer management and coverage measures on soil nutrients

In this experiment, the content of NO₃ ⁻ -N in the soil during the flower bud differentiation period, fruit filling period and maturity period of sugar apple was the highest under W2F3A1 treatment, which was attributed to the influence of multiple factors. Under the condition of no mulching, the nitrogen application rate increased, and the minerals in the soil remained in the form of NO₃ ⁻ -N, which promoted the accumulation of N elements [32]. However, maintaining soil moisture at a relatively low level limited the formation of a sufficient water potential gradient to leach nitrate nitrogen beyond the root zone, significantly reducing nitrogen leaching losses [33]. In 2022, applying straw as mulch yielded significant enhanced NO₃ ⁻ -N levels, but in 2023, the effect was lower, which was related to the lag of straw decomposition and the accelerated nutrient leaching in rainy climates. In contrast, the highest NO₃ ⁻ -N content in 2023 during the flowering and fruit setting period of *Annona squamosa* was in W1F3A3 treatment due to the lower rainfall in orchards in 2022 than in 2023. The risk of leaching was reduced by moderate deficit irrigation under high fertilization, and the NO₃ ⁻ -N was accumulated by reduced evaporation and runoff from straw mulching, but the initial decomposition is slow, and the nitrogen contribution of straw decomposition to the soil in the first year is limited [34]. After two consecutive years of field trials in 2022 and 2023, the organic matter covered with straw released nitrogen by decomposition, and combined with the residual effect from the previous year, it significantly increased the nitrogen content [35]. Under high fertilization and no mulching conditions, moderately deficit irrigation reduced soil moisture and significantly reduced the rate of P-related hydrolysis and fixation reactions, resulting in more P in its effective form [36]. This is consistent with the results of our study of increased soil available phosphorus content under W2F3A1 treatment. 2022 data indicated a significant effect of mulch application on soil AK enhancements in grassy areas. Due to the low rainfall in 2022, the potassium content in raw grass is high, and the decomposition rate is fast, while adequate irrigation promotes the dissolution and migration of potassium ions to the rhizosphere [37]. while in 2023, the rainfall is heavy, and the straw mulch enters the peak decomposition period in the second year, releasing potassium elements, which is conducive to potassium accumulation [38]. Therefore, the highest AK content values were reached in 2022 and 2023 under different treatments, which is consistent with previous studies. The interaction of water and fertilizer enhances soil structure stability markedly. Straw mulching significantly enhanced soil nutrient availability and uptake due to increased organic matter content, reducing soil bulk density and optimizing soil aggregate stability, thereby optimizing soil structure and activating microbial functions [39].

### 4.2. Water and fertilizer management and mulching measures affect soil enzyme activity

The results showed that the highest soil urease activity and catalase activity under W2F2A3 treatment in 2022 and 2023 depended on the multifactor interaction between water and fertilizer cover. This is because urease activity is higher under aerobic conditions, and moderate deficiency irrigation not only provides sufficient water to maintain microbial movement, but also ensures sufficient oxygen to promote urease activity. Whereas moderate fertilization provides sufficient urea substrate to promote the production of more urease by microorganisms [40,41]. Straw mulching provides sufficient carbon source, and in order to maintain a appropriate C/N ratio, the demand for nitrogen strongly stimulates microorganisms to secrete urease to decompose urea to obtain a fast-action nitrogen source [42]. Moderate moisture content can maintain the gas-liquid balance in soil pores, protect the spatial structure of enzymes, and facilitate catalase contact with soil substrates [43]. The nutrients input from fertilization are synergistically supplemented with the organic nutrients from straw decomposition to maintain microorganisms in a state of high metabolic activity and promote the continuous synthesis of catalase [44]. Previous studies have indicated that under short-term experiments, straw decomposition is insufficient, grass root exudate can stimulate microbial growth and metabolism, and low irrigation and fertilization rate reduce soil nutrient P leaching, providing a more sufficient substrate for phosphatase activity [45]. This is the reason why the phosphatase activity of *Annona squamosa* was the highest during the whole growth period in 2022 under low water, low fertilizer and fresh grass cover. The entropy effect of phosphatase activity in 2023 was best achieved under moderate irrigation and fertilization and straw mulching because under the long-term effects of water and fertilizer management and mulching measures, straw mulching residues provided sufficient metabolic substrates for microorganisms to promote their nutrient uptake, and appropriate water and fertilizer conditions improved the dissolution and mineralization rate of crop rhizosphere organic nitrogen, increasing the content of mineralized nitrogen in the soil and accelerating the secretion of N and P cycle enzymes [46].

### 4.3. Water and fertilizer management and mulching measures affect soil microecology

In this study, moderate irrigation, fertilization, and straw mulching treatments significantly enhanced the α-diversity of rhizosphere soil microorganisms, as evidenced by higher Chao1, Shannon, Simpson, and PD_whole_tree indices compared to other treatments. The results showed that the Chao1 index and Shannon index were higher under W1F2A2 treatment, because fertilization had no significant effect on bacterial diversity under low water and low fertilizer conditions [47]. When irrigation is insufficient, wetness and drought alternately influence carbon and nitrogen availability, stabilizing the microbial community. Root exudate promotes microbial growth and enhance species abundance [48]. The higher index of A3 treatment in the PD_whole_tree index is due to reasonable water and fertilizer conditions that provide carbon, nitrogen and energy for microorganisms by regulating soil moisture and nutrient status and making full use of the interaction of various elements [49], while straw mulching stabilizes soil temperature, increases soil NO₃ ⁻ -N, provides a good environment for soil microorganisms, and enhances soil microbial retention [50]. The relative abundance of *unclassified_Vicinamibacteraceae* in W1F2A2, W2F1A2, and W3F3A2 treatments was due to the release of secretions around the root system by grasses, which provided abundant nitrogen and carbon sources for *Vicinamibacteraceae* and promoted microbiota growth [51]. *Bryobacter* has oligotrophic bacteria, can efficiently use trace nitrogen sources in the soil to quickly obtain nutrients, provide oxygen for microorganisms with sufficient moisture, and release organic acids from straw decomposition, which is conducive to *Bryobacter* becoming the dominant flora in W3F1A3 treatment [52]. As a common agricultural measure, mulching measures have a significant impact on soil microbial community structure. In this study, the soil microbial community structure was significantly different between mulching and no mulching. Compared to no mulching, cover crops enhanced the availability of C and N substrates by altering the contact between residues and soil microorganisms, which was beneficial for microbial growth and enhanced soil microbial community structure [53]. In this study, *unclassified_Chloroflexi*, *Pseudomonas* and *RB41* showed high relative abundance in W2F2A3 treatment, which was the result of the multi-factor interaction of water and fertilizer mulching. Straw mulching promotes the nitrogen metabolism capacity of soil microorganisms, reduces oxygen emissions, increases *unclassified_Chloroflexi* abundance, promotes sustainable soil production, and improves crop productivity [54]. As a beneficial fungus in the rhizosphere of plants, *Pseudomonas* not only helps to improve the plant’s ability to absorb water and nutrients adequately, but also its biological nitrogen fixation capacity can increase crop yields [55]. Moderate deficit irrigation can accelerate the decomposition efficiency of straw and shorten the cycle of *RB41* to obtain carbon sources. Concurrently, straw mulching can reduce soil nutrient leaching and maintain the nutritional stability of the medium-fertilizer environment for the survival of *RB41* [56]. *unclassified_IMCC26256* enriched in acid-tolerant soils, straw mulching reduced soil CO_2_ to alleviate soil acidification, *unclassified_IMCC26256* relative abundance decreased [57], which is the reason for the decrease in relative abundance under W2F2A3 treatment. The relative abundance of *Haliangium* decreased under fertilization treatment, which may be due to the continuous application of chemical fertilizers reducing the abundance of potential biocontrol and growth-promoting bacteria, and weakening the soil nitrogen cycle, thereby inhibiting the relative abundance of *Haliangium* in soil [58].

It is worth noting that among the key taxa treated in this study, many unclassified units such as *unclassified_Vicinamibacterales*, *unclassified_Gemmatimonadaceae*, etc., may drive the response of soil microbial communities to water-fertilizer cover coupling at the family or object level, rather than at the level of individual genera. Due to the limitations of current microbial databases, many taxa in specific environments may not be accurately classified at the genus level, and the specific functions of these unclassified communities will be further elucidated in the future. It should also be pointed out that although this study has clarified the regulatory effects of optimized agronomic measures on the soil microbial community structure in passion fruit orchards, it is limited by the existing data and has not yet directly verified the actual ecological functions of dominant microbial groups or their causal relationship with ecosystem functions, leading to a lack of direct evidence to support the interpretation of mechanisms behind microbial community changes. Therefore, in the future, we will further conduct experiments to systematically verify the functional roles of microbial groups (especially unclassified units), clarify their correlation mechanisms with soil nutrient cycling and crop growth and development, and ultimately refine the scientific framework that demonstrates how water-fertilizer-cover coupling measures enhance ecosystem functions by regulating the microbial community.

### 4.4. *Annona squamosa* crop performance and quality influenced by water, fertilizer, and mulching measures

Balanced water and nutrient pairing is crucial for optimizing fruit tree yield and quality [59]. The experimental results showed that in 2022 and 2023, the output of *Annona squamosa* was higher when using the W2F2A3 and W2F3A1 methods, respectively. This is because under the synergistic effect of irrigation, fertilizer, and mulching, the soil fertility of the rhizosphere of *Annona squamosa* is enhanced, and the good soil nutrient status is conducive to the health of fruit trees, which contributes to an increase in *Annona squamosa* production. A lack of adequate irrigation and fertilizer deficit irrigation can inhibit the overdevelopment of fruit tree nutritional organs and the enhancement of organic substance buildup that are transferred to the fruit. However, severe water shortage will cause the cell walls of fruit cells to harden, inhibit the expansion of pulp cells, and stimulate the production of cellulose, thereby reducing the fruit load and lowering the single fruit weight and yield [60,61]. In 2023, compared with 2022, the fruit output was the highest under low fertilizer and no mulching, which was due to the extension of the fertilization period and the enhanced ability of soil nutrients to accumulate. Grass mulching competes with fruit trees for nutrients, but the improvement in yield was not significant. Under long-term straw mulching, its thermal regulatory effect may outweigh the entropy retention effect, thereby suppress microbial activity and decelerate organic matter decomposition [62]. The study revealed that *Annona squamosa* exhibited the highest content of soluble solids under conditions of medium water-fertilizer application combined with straw mulching. Sustainable water and fertilizer management can promote rapid fruit growth from the late filling stage to ripening, maintain photosynthetic efficiency, enhance photoassimilate accumulation, and consequently increase soluble solids content (SSC) to improve fruit quality [63]. Straw mulching treatment was most beneficial for total soluble solids (TSS) accumulation, not only reducing fruit acidity but also increasing fructose content, thereby providing positive feedback for vegetative growth and fruit quality [64]. In this study, the two-year data showed that the soluble sugar content of *Annona squamosa* under straw mulching condition was higher than under the grass and the no-mulching conditions, and it significantly increased the sugar-acid ratio, which aligns with findings from earlier research. Among them, the content of vitamin C in *Annona squamosa* was most abundant under moderate irrigation and mulching measures, because excessive irrigation and high soil humidity will dilute soluble solids and acids, inhibit vitamin C synthesis, and affect the flavor and nutritional quality of the fruit [65]. Applying organic mulch boosted the leaf’s nutrient levels and enhanced the soil’s nitrogen accessibility, all of which spurred the growth of fruit trees. Moreover, using straw as a decomposition base encouraged the production of vitamin C in the fruit [66].

## 5. Conclusion

Through analyzing the regulatory effects of irrigation, fertilization and mulching measures on soil quality and yield characteristics in the root zone of *Annona squamosa*. The study indicated that the W2F3A1 treatment enhanced soil nutrient supply capacity and improved soil quality; The optimal combination treatment W2F2A3 (mildly deficit irrigation, moderate fertilization, straw mulching) demonstrated superior performance, enhancing key soil enzyme activities, increasing beneficial microbial relative abundance, and optimizing microbial community structure. Improved soil quality provided a favorable rhizosphere environment for *Annona squamosa* growth, thereby enhancing yield and quality. Through PCA-GRA algorithm evaluation, the W2F2A3 treatment was confirmed as the optimal scheme. This study elucidated the key mechanisms by which water-fertilizer-mulch synergistically optimized soil quality. The proposed technical approach provided practical guidance for enhancing soil quality, improving ecological conditions, achieving coordinated yield and quality enhancement, and implementing sustainable management in the green cultivation of Yunnan *Annona squamosa*.
